# A case-based review of adult-onset craniopharyngioma

**DOI:** 10.3389/fendo.2025.1527161

**Published:** 2025-05-15

**Authors:** Scott Meyer, Shruti N. Shah, Kristen Dancel-Manning, Yuxiu Wang, Matthew Young, Nidhi Agrawal

**Affiliations:** ^1^ Department of Medicine, NYU Langone Health, New York, NY, United States; ^2^ Department of Endocrinology, NYU Langone Health, New York, NY, United States; ^3^ Department of Pathology, NYU Langone Health, New York, NY, United States; ^4^ Department of Radiology, NYU Langone Health, New York, NY, United States

**Keywords:** craniopharyngioma, adult craniopharyngioma, papillary craniopharyngioma (PCP), adamantinomatous craniopharyngioma, mini review, literature review, case-based

## Abstract

Craniopharyngiomas are histologically benign central nervous system tumors derived from embryonic epithelial cells of Rathke’s pouch. The disease demonstrates a bimodal age distribution, occurring most often in patients 5-14 and 50-74 years of age. Common comorbidities include hypopituitarism, hypothalamic obesity, sleep apnea, visual impairment and neurocognitive disturbances. There are several key differences in the presentation, tumor characteristics and clinical outcomes between age groups. Childhood craniopharyngiomas are mostly adamantinomatous and often present as larger tumors with worse functional outcomes such as rates of obesity and neurological deficits. Adults experience similar but slightly adjusted rates of comorbidity with both the adamantinomatous and papillary subtypes. This review presents a case-based discussion of adult craniopharyngiomas, focusing on recent literature regarding their presentation, pathology and pathogenesis, diagnosis, treatment and long-term sequelae.

## Introduction

Although craniopharyngioma is often considered a pediatric disease, up to half of cases are diagnosed in adulthood. These central nervous system tumors occur at a rate of 0.5-2 cases per million person-years (1). Despite their benign classification, they cause significant morbidity for afflicted patients prompting ongoing research efforts and review. Historically, adult-onset craniopharyngioma has been less well characterized than childhood craniopharyngioma. There are subtle differences in the disease script of adult versus childhood craniopharyngioma to note though. Namely, the papillary (PCP) variant is almost exclusively found in adults while children are almost principally affected by the adamantinomatous (ACP) variant (2). Considering this, there are specific elements of diagnosis, treatment, and ongoing sequelae to consider when encountering an adult-onset case of craniopharyngioma. Furthermore, over the last decade there has been progress in treatment approaches including refined surgical techniques, adjuvant radiation methods and targeted immunotherapies, some of which are unique to the adult cohort.

Here, we present two cases of adult-onset craniopharyngioma that were treated at our institution, illustrating specific features, challenges and therapeutic opportunities for adult craniopharyngioma patients. In doing so, we also review the recent literature focusing on this specific patient population. Though not a systematic review, this article was generated through a methodical search of literature listed within the PubMed database over the last 15 years. Articles containing “adult” and “craniopharyngioma” in the title, or articles containing the string of text “adult craniopharyngioma”, “adult onset craniopharyngioma” or “adult-onset craniopharyngioma” were included in initial review. References that these publications cited were also thoroughly reviewed to gather further information. In rare cases when this method failed to identify certain information that would be of use, sparse use of targeted PubMed searches to supplement the article was utilized.

### Case 1

A 54-year-old female presented to the ophthalmology clinic with blurry vision for a month. Visual field testing revealed a right homonymous defect, an incongruous left visual field and bilaterally decreased acuity. MRI of the pituitary with and without gadolinium revealed a 2.5-cm suprasellar mass with solid and cystic components causing mass effect on the optic chiasm and hypothalamus. The patient underwent an endoscopic endonasal transsphenoidal resection of the tumor, and pathology confirmed WHO 1 ACP. Postoperative workup revealed a morning cortisol of 1.8 mg/dL, a free-T4 of 0.5 ng/dL and concerns for vasopressin deficiency, for which she was started on steroid replacement, levothyroxine and desmopressin. Imaging confirmed residual tissue near the right hypothalamus and mammillary bodies. Adjuvant radiation therapy using fractionated external beam radiation was considered, but the patient opted not to pursue radiation treatment out of concerns of potential adverse events. Serial MRIs over the last 5 years have not yet demonstrated significant disease progression.

### Case 2

A 33-year-old male presented with worsening memory and vision changes over the last few months. Visual field testing demonstrated a right homonymous hemianopsia, and MRI of the pituitary confirmed a 3-cm suprasellar cystic mass extending into the third ventricle with mass effect on the hypothalamus and optic chiasm. At an outside institution, the patient underwent a craniotomy and cyst drainage with initial pathology erroneously diagnosing a Rathke cleft cyst. Six months after surgery, he re-presented with worsening vision and a recurred 3.6-cm suprasellar cystic mass. He underwent a gross total resection of the tumor via an endoscopic endonasal approach. Pathology confirmed BRAF+ PCP. His course was subsequently complicated by hypothalamic obesity and panhypopituitarism, which has been managed with steroid replacement, levothyroxine, desmopressin, growth hormone and gonadotropins. Subsequent MRIs have been stable with no evidence of recurrence.

## How do adult-onset craniopharyngiomas present?

Craniopharyngioma presentation is often insidious with symptoms that can be difficult for patients and clinicians to identify early. In the above two cases, both patients presented with concerns about subacute changes in their vision. Our ACP patient indicated to her ophthalmologist that over the last month her vision appeared blurry even when wearing her recently prescribed glasses. Although her fundoscopic exam was normal, a thorough visual field exam noting significant defects prompting her MRI and ultimate diagnosis. For our PCP patient, he entered our health system through the emergency department as he felt like his visual deficits were not being addressed promptly enough by his other outpatient providers. He had an outpatient neurosurgery appointment the day prior to go over these concerns, but after feeling as though it was starting to deteriorate further presented to the emergency department for additional input. Once admitted he underwent further imaging, resection, and was ultimately diagnosed with craniopharyngioma rather than his previously thought Rathke cleft cyst. In adults, vision changes are one of the most frequent symptoms experienced at the time of diagnosis. Studies site the most frequent adult complaints as visual impairments (40-84%), menstrual abnormalities (57% of women), headaches (47-56%), decreased energy (32-48%), nausea/vomiting (16.7-26%), weight gain (13-18.9%) and polyuria (14.2%) (3–7). There is significant overlap in symptomatology between adults and children but there are some differences in the rates of these symptoms. A study by Catina et al. to elucidate differences in presenting symptoms between children versus adults noted statistical differences for nausea/vomiting (57.8% vs 16.7% of children versus adults respectively), photophobia (21.7% vs 5.6%), diabetes insipidus (28.5% vs 8.3%), growth hormone (GH) deficiency (66.8% vs 17.1%), decreased visual acuity (44.1% vs 67.6%) and decreased peripheral vision (51.6% vs 71.4%) (6). They also noted a slightly higher rate of headaches in children versus adults though not to a significant level. A pooled meta-analysis by Pang et al. found similar results: rates of visual deficits (54.51% vs 74.2%) and endocrinopathies (48.88% vs 57.4%) were lower in children vs adults, respectively. However, they also noted significantly higher rates of headaches (60.04% vs 40.3%, *p* < 0.001) and cognitive impairment (6.76% vs 19.48%, *p* < 0.001) in children vs adults, respectively (8). A potential explanation for such differences may be that pediatric cases present with large tumors (>3 cm, 54.1% vs. 31.8%, *p* < 0.001) (9) more often than do adult cases and, in turn, have higher rates of symptoms related to increased intracranial pressure. Ultimately, the most common symptom among adults, as demonstrated with our two patients, is insidious visual worsening.

### Pathology of ACP and PCP

ACP and PCP have classically been distinguished using histopathologic features. ACPs are composed of well-differentiated epithelium arranged in cords, lobules and nodular whorls, bordered by palisaded columnar epithelium. The tissue often features cystic cavities containing cell debris, fibrosis and cholesterol; anucleate eosinophilic “ghost cells”; and distal finger-like protrusions that invade nearby structures (10–12). Conversely, PCPs are composed of monomorphic sheets of well-differentiated squamous epithelium with fibrovascular cores and lack many of the more distinctive structures found in ACP. In some PCP cases, there are areas of ciliated epithelium and PAS+ goblet cells that can resemble the architecture found in Rathke’s cleft cyst (10); for the PCP patient described above, this finding was likely what led to the initial inaccurate Rathke’s cleft cyst diagnosis. By examining additional tissue gathered from his second resection and performing additional immunostains pathologists were able to state that his case was more consistent with PCP. Beyond histology, the two variants have several well-established differences, especially with regard to their epidemiology. While ACP typically presents in children, it can also occur in adults, with a second peak of incidence at 50-74 years old. PCPs, on the other hand, occur almost exclusively in adults aged 40-55 years (2, 3, 10). PCP patients tend to present with smaller tumors than ACP patients (8.7 ± 12.2 cm^3^ vs. 19.7 ± 17.9 cm^3^, respectively; *p* < 0.05) but without any significant differences in thalamic involvement or pituitary stalk compression (5). While our PCP patient’s tumor was larger than our ACP patient’s, it was potentially a particularly aggressive one as it was recurrent and had already been operated on several years prior. Regarding disease sequelae, PCP has higher rates of associated diabetes insipidus (36.8% vs 16.7%; *p* < 0.05) and lower rates of obesity and weight gain (0% and 5% vs 38% and 26%) compared to ACP, but overall clinical presentation between the two disease entities are largely analogous (5).

It is now established that the WNT signaling pathway is instrumental in ACP pathogenesis through the CTNNB1 gene encoding β-catenin. Genetic analyses have noted an activating mutation in exon 3 of CTNNB1 in up to 96% of cases, and many centers now perform immunostaining or sequencing of β-catenin for diagnostic purposes (13–17). In our ACP patient, pathology reports indicated finding rare but definitive staining of β-catenin. The mutations are believed to increase the half-life of β-catenin, resulting in nucleocytoplasmic accumulation and increased pro-oncogenic signaling. Specifically, transcriptomic studies have noted the upregulation of EGFR and SHH signaling pathways in ACP, which are proposed to contribute to growth and migration (18–20). The molecular background of PCP was largely unknown until exome sequencing studies performed in 2014 noted high rates of the BRAF V600E mutation. This proved useful in case 2 when a positive BRAF V600E immunostain followed by a confirmatory BRAF DNA sequencing test helped to lead to the diagnosis of PCP rather than ACP or Rathke’s cleft cyst. The majority of literature appears to suggest that the mutation is exclusively found in PCP cases, with Brastianos et al. observing the BRAF V600E mutation in 95-100% of PCP cases and CTNNB1 alterations in 92-96% of ACP cases without overlap (17). A recent small study noted concurrent BRAF V600E mutation and CTNNB1 alterations in 2 of 14 ACP cases (21), but this finding has not yet been replicated, and a follow-up study done specifically to address this question identified BRAF mutations in 33 of 33 PCP cases and 0 of 79 ACP cases (10). This study also revealed that no β-catenin accumulations were found in PCP immunostaining. Ultimately, whether BRAF mutations might coexist with CTNNB1 mutations remains an open question, but current literature suggests that their mutational etiologies of PCP and ACP are fundamentally different and distinct.

Helping to further distinguish between ACP and PCP, recent proteogenomic studies have shown how their overall gene expression profiles differ. Holsken et al. looked at gene expression in a small subset of 18 ACP and 10 PCP samples and at CpG methylation profiles of 482,421 sites in 25 ACP and 18 PCP samples. Despite the low number of samples, when analyzing the gene expression profiles, unsupervised consensus clustering resulted in two distinct and stable clusters separating ACP and PCP samples. Furthermore, when looking at the methylation data, similarly unsupervised clustering with principal component analysis was able to segregate ACP and PCP cases (10). The methylation profiles of ACP in pediatric and adult cases were similar, highlighting a similar pattern of molecular mimicry despite differences in other disease traits between childhood and adult-onset ACP. The ongoing research into the molecular underpinnings of these tumors promises to further refine diagnostic criteria and treatment strategies, offering hope for better management of this challenging condition.

### Imaging findings in ACP and PCP

Radiological assessment plays an imperative role in initial craniopharyngioma diagnosis, characterization and management for children and adults alike. MRI is the imaging modality of choice for differentiation from other sellar masses and typically demonstrates a mixed solid-cystic or solid lesion in the suprasellar cistern (22–24). In both of our cases, the tumors were confined to the suprasellar region, but about 25% of cases have an intrasellar component, and rare cases (<5%) are purely intrasellar (25). ACPs appear lobulated on imaging with a greater frequency of calcifications compared to PCPs, which tend to be more spherical and predominantly solid (though still frequently with a mixed solid-cystic component) (26, 27). The MRI report of the ACP patient described her tumor as a “suprasellar, cystic and solid mass inseparable from the pituitary infundibulum with heterogeneous T1 and T2-weighted signal features. The majority of the cystic component is intrinsically T1 bright suggesting hyperattenuation contents or intralesional hemorrhage … [though] signal voids within the cystic component of the mass [are] suggestive of calcification.” T1-weighted imaging can prove useful for distinguishing ACP and PCP, as ACP often has T1 hyperintense cystic components due to high protein content, whereas PCP tends to be hypointense (27). This led to the radiologist commenting in the MRI impression that they had a higher suspicion of ACP over PCP for this patient. Regardless of subtype, most craniopharyngioma cases demonstrate a cystic component. In a study of 38 craniopharyngioma cases (30 ACP and 8 PCP), only one PCP case did not demonstrate cystic changes on T2-weighted MRI (26). Both our ACP and PCP patient demonstrated cystic components. Due to the overlap in appearance, distinguishing ACP from PCP in adults with imaging alone remains an ongoing challenge for radiologists. On CT imaging, calcification has an approximately 83.3% sensitivity (25 of 30) and 100% specificity (8 of 8) for distinguishing ACPs from PCPs (26). However, other studies have found calcifications on imaging for PCPs, suggesting that calcification is not truly a 100% specific marker for ACPs (27). On MRI, T1 hyperintensity had a sensitivity of 73.3% (22 of 30) and specificity of 75% (6 of 8) for distinguishing ACPs from PCPs (26). In recent years, several attempts have been made to discriminate ACP from PCP using machine learning analysis of MRI (24, 28, 29). Two models, a random forest classifier model developed from 44 patients and a SVM classifier from 99 patients, achieved AUC values of 0.89 (with a sensitivity of 0.89 and specificity of 0.85) and 0.92, respectively (28, 29). A fully automated classification without radiologic pre-segmentation was also attempted, achieving an AUC of 0.838 (with a lower sensitivity of 0.608 and a specificity of 0.845). The smaller sample size and MRI quality were thought to be limiting factors in this study (24). Although ongoing work is needed to improve imaging diagnostic abilities in light of the heterogeneous nature of adulthood craniopharyngiomas, it remains a powerful tool for characterization and management.

### Management of adult-onset craniopharyngioma

Adult-onset craniopharyngioma poses challenges in treatment due to its intricate anatomical location and potential impact on critical neurological and endocrine functions. The limited availability of data, coupled with the dearth of comparative clinical trials and prospective studies over the past decade, has hampered the development of concrete treatment protocols. Consequently, the management strategies for this condition largely rely on retrospective analyses and expert consensus, highlighting the need for more robust clinical research to optimize patient outcomes.

The primary treatment modality for adult-onset craniopharyngioma is surgical resection, aiming for maximal tumor removal while preserving neurological function. Surgical approaches can be categorized broadly into transcranial and transsphenoidal techniques, the choice of which is contingent upon the tumor location and relationship to surrounding structures (30). The transcranial approach had been the standard approach for surgeons for many decades; however, with ongoing technical advances, the transsphenoidal approach via extended endonasal endoscopy has been increasingly utilized as was done for both the ACP and PCP cases described above (31). Sellar and suprasellar tumors are well suited in particular for the endoscopic endonasal approach, with limitations arising for tumors with third ventricular involvement and tumors with extension lateral to the internal carotid arteries (32, 33). GTR was achieved in the PCP patient described in Case 2 whereas the ACP patient in Case 1 had a STR due to the tumor’s abutment to the hypothalamus and mammillary bodies. Gross total resection (GTR) is often pursued to minimize recurrence rates and improve long-term tumor control, as compared to the more conservative approach of subtotal resection (STR) combined with adjuvant radiation therapy (RT). A study by Godil et al. found that the 5-year recurrence-free survival rate was 75.0% after GTR compared to 25.0% after STR; the time to recurrence after GTR was 30.2 months versus 13 months after STR; and patients with GTR had a lower rate of visual deterioration and higher rate of return to work or school compared with those with STR (34). Fortunately for our ACP patient, despite only undergoing a STR, seven years out from her procedure she has not had recurrence or progression of disease. **Table 1** summarizes surgical studies conducted over the last 15 years that directly discussed achieved GTR rate and subsequent comparative outcomes in GTR versus STR patients (7, 9, 31, 34–57). Consistent with the findings of Godil et al., a central theme across these studies was that GTR patients had lower rates of recurrence compared to their STR counterparts. Although not all of the studies performed statistical analysis on recurrence, those that did often found the differences to be statistically significant. Further, when STR with adjuvant RT was isolated as a group for analysis, recurrence rates frequently fell somewhere in between those of the GTR and the STR-alone cohorts. The majority of these studies included a mixed adult and patient cohort. However, the findings within the eight adult-only studies largely aligned with those of the mixed-age studies. Despite the improvement in recurrence among GTR patients compared with STR patients, the intricate anatomy of the sellar and suprasellar regions often necessitates a balance between complete resection and the risk of morbidity as with Case 1. Particularly, GTR has been associated with higher risks of diabetes insipidus, visual deficits, panhypopituitarism and other endocrinopathies when compared to STR plus RT (34, 51, 58, 59). Reassuringly, recent advances in neuroimaging, intraoperative neuro-navigation and endoscopic techniques have enhanced the safety and efficacy of these neurosurgical procedures (60, 61).

**Table 1:** Primary surgical studies conducted over the last 15 years with comparative data regarding GTR versus STR^a^.

Author (reference)	Sample size	Age	Subtype	Surgical Approach	GTR Proportion	% Use of Adjuvant RT (GTR vs STR)	Statistically significant findings (GTR vs STR)	Recurrence (GTR vs STR)
Iglesias et al. (35)	53	65-83 (72.3 ± 5.2)	51% ACP, 45.1% PCP^a^	Transcranial (52.8%), EET (41.5%), Microscopic transphenoidal (5.7%)	45.30%	18.9% of all cases	Diabetes insipidus at ~47 months of follow up (89.5% vs 57.1%, p=0.017)	4% GTR, 17% STR + NTR ^b,c^
Sadashivam et al. (36)	95	37.7 ± 13.6	88% ACP, 12% PCP	Pterional craniotomy (85%), EEN (4%), other (11%)	63%%	N/a	Tumor recurrence (14% vs 41%, p=0.01)	14% GTR, 41% STR
Park et al. (37)	64	46.7 ± 12.7 (20-75)	75% ACP, 25% PCP	Transcranial (48.4%), transsphenoidal (51.6%)	69%	12.5% (0% GTR, 66% of STR)	Kanofsky peformance scale higher in GTR than in STR (p=0.009)	25% GTR, 62.5% STR+RT, 75% STR
Dandurand et al. (38)	22	46.7± 15.2	55.6% ACP, 44.4% PCP	Transcranial (86.4%), endonasal (13.6%)	46%	9% (10% GTR, 8% STR)	N/a	17% GTR, 27% STR+RT, 45% STR ^d^
Bosnjak et al. (39)	8	63 (47-73)	100% ACP	EES (100%)	75%	0%	N/a	N/a
Lee et al. (40)	90	43.3 (21-71)	N/a	Pterional, transcallosal, frontobasal, and transsphenoidal	72%	15.6% (6% GTR, 40% STR)	Progression free survival (93.8 months for GTR group vs 25.9 months for STR group vs 44.3 months in the STR + RT/SRS group, p=0.030)	32.8% GTR, 73.3 STR
Kim et al. (41)	146	41.4 (18-75)	65% ACP, 30% PCP, 5% “Mixed”	Unilateral pterional (77%), interhemispheric (11%), transsphenoidal (10%)	36%	56% of STR cases	Long term visual defecits (51% STR vs 15% of GTR and 19% of STR w/adjuvant, p<0.001), recurrence rate (56% STR, 19% GTR, 14% STR w/adjuvant p<0.001)	56% STR, 19% GTR, 14% STR w/ART
Jung et al. (42)	41	45.8 (17-65)	73.2% ACP, 26.8% PCP	Subfrontal or pterional (66%), basal bifrontal (12.8%), transcollosal transventricular (10.6%), transsphenoidal (6%), transcallosal/pterional combined (2.1%)	77%	10% of all cases	Recurrence-free survival rate higher for GTR than STR (p=0.023)	23.3% GTR, 33.3% STR
Lee et al. (43)	81	42 (15-79)	76.5% ACP, 23.5% PCP	Transcranial (96.3%), transphenoidal (3.7%)	86.60%	20% of near/subtotal resection cases	Overall postoperative complication rate (45.5% vs 22.5%, p=0.053)^e^	9.9% GTR, 14.3% NTR, 66.7% STR
Lopez-Serna et al. (44)	153	32.4 (16-77)	83.6% ACP, 16.3% PCP	Transcranial (84%), transphenoidal (microscopic transeptal and purely endonasal) (16%)	30.46% (and NTR in an additional 21.85%)	N/a	Lower overall survival rate at 5, 10, 15, and 20 years for GTR/NTR vs STR/PR (p<0.05)^f^, lower TSH/T3/T4 in GTR/NTR group vs STR/PTR group (p<0.0001) lower cortisol levels in GTR/NTR vs STR/PTR (p<0.01)	10.86% GTR, 41.07% NTR/STR
Godil et al. (34)	44	55.9 ± 20.2	74.2% ACP, 25.8% PCP	Extended transsphenoidal transplanum (95.5%), transclival (4.5%)	77.30%	2.9% for GTR, 70% for STR	5-year PFS 75.0% after GTR and 25.0% after STR (45% in STR + RT; p < 0.001), TTR after GTR was 30.2 months, 13 months after STR (5.8 months in STR + RT; p < 0.001), GTR had a lower rate of visual deterioration (2.9%) and higher rate of return to work/school (91.2%) compared with patients with STR (visual deterioration 20%; p = 0.04 and return to work/school 60%; p = 0.02), GTR group had a higher rate of permanent DI (85.3%) than did the STR group (50%; p = 0.02)	20% GTR, 70% STR
Yadav et al. (45)	44	42 (8-65)	N/a	Endoscopic endonasal extended transsphenoidal (100%)	59.10%	22% of STR & partial cases	N/a	4% GTR, 18% STR, 43% PR
Lehrich et al. (9)	3638	41	N/a	N/a	13%	14% for GTR, 31.5% for STR	N/a	N/a
Wijnen et al. (46)	128	19 (8-42)	N/a	Transsphenoidal (39%), subfrontal (35%), pterional (23%), transcallosal (2%) and supra-orbital (1%)	20%	57% for STR	N/a	85% (not stratified by GTR vs STR)
Shi et al. (47)	1054	23.8 (9mo-77yo)	80% ACP, 20% PCP	Pterional craniotomy (21.5%), unifrontal basal interhemispheric approach (64.8%), other craniotomy (13.7%)	89.60%	N/a	N/a	13% GTR, 47.3% STR + RT, 94.2% STR
Park et al. (48)	116	43.8 (14-74)	71% ACP, 27% PCP, 2% Undetermined	Endoscopic endonasal (100%)	46%	29% of NTR and STR cases	Improved recurrence rate survival for GTR than NTR or STR alone (excluding adjuvant RT patients) (p=0.0116)	9.3% GTR, 18.2 NTR, 27.8% STR
Dhandapani et al. (49)	57	45 (5-85)	96% ACP, 4% PCP	Endoscopic endonasal extended transsphenoidal (100%)	87%	22% of GTR and NTR cases, 75% of STR cases	Tumor recurrence (p=0.02)	4% GTR+NTR, 33% STR
Lo et al. (50)	123	30 (2-80)	N/a	N/a	15%	58% of STR cases	N/a	N/a
Koutourousiou et al. (31)	64	39.8 (4-82)	N/a	Endoscopic endonasal extended transsphenoidal (100%)	37.50%	72.7% of all cases	N/a	25% GTR, 40% STR
Schoenfeld et al. (51)	122	30 (11-52)	65% ACP, 12% PCP, 23% unknown	Endoscopic endonasal (27%), craniotomy (73%)	27%	9% of GTR, 54% of STR	Improved progression free survival for GTR and STR+RT than STR alone (p < 0.001 for both), rates of DI(56.3 GTR vs. 13.3% STR + XRT, p < 0.001), rates of panhypopituitarism (54.8 GTR vs. 26.7% with STR + XRT, p = 0.0.014)	46% (not stratified)
Zacharia et al. (52)	644	0-80+	29.7% ACP, 8% PCP, 62.1% unknown	N/a	47%^g^	22% of all cases	N/a	N/a
Leng et al. (53)	24	43.6 ± 20.3 (5-82)	N/a	Endoscopic endonasal (15% transsellar, 85% extended)	69%	17% GTR, 75% NTR/STR	N/a	0% GTR, 75% NTR and STR
Zhao et al. (54)	151	29.0 ± 17.3 (1-68)	N/a	95% Pterional, 4% transcallosal, other transcranial 1%	70.30%	35% GTR, 71% STR	Permanent diabetes insipidus (32.1 GTR, 15.6% STR, p=0.037), improved survival and recurrence rate for GTR vs STR (p<0.01)	10.1% GTR, 8.1% GTR + RT, 92.3% STR, 31.3% STR+RT
Mortini et al. (7)	112	33.3 ± 1.8 (6-78)	92.9% ACP, 7.1% PCP	Transphenoidal (32.1%), transcranial (67.9%)	71.60%	7% of all cases	5-year recurrence-free survival (85.6% GTR, 46.3% STR, p<0.001)	24.5% (not stratified)
Pekmezci et al. (55)	80	43.5 (0-79)	86% ACP, 11% PCP, 2% unknown	Frontotermporal (57%), bifrontal craniotomy (15%), transphenoidal (25%), n/a (3%)	13.20%	44% of all cases	N/a	44% GTR, 57% STR
Jane Jr et al. (56)	12	50.8 (29–76)	58% ACP, 33% PCP, 8% unknown	Endoscopic transsphenoidal (100%)	41.67%	N/a	N/a	50% STR
Kawamata et al. (57)	55	26.6 (2–68)	N/a	Transcranial (n/a) and transphenoidal (n/a)	22%	60% of all cases	N/a	N/a

a Meta-analysis were not included nor were studies with only pediatric patients.

b An additional 3.9% of tumors were classified as “mixed” in histology.

c In this study, near total resection (NTR) was defined as the removal of more than 90% of the tumor volume. Other studies listed in the table varied slightly in their definition of NTR.

d For this study, only cases of recurrence that required additional surgeries were noted in this data point.

e Recurrence rates were from a meta-analysis the paper performed rather than the isolated case series data.

f A non-significant but of note finding was all surgery related deaths, ICH, and cerebral infarcts were isolated to GTR group.

g Partial resection (PR) was defined as <60% tumor removal.

h Only 462 of the patients underwent surgical intervention. The listed rate of GTR (47%) is only considering the patients that underwent surgery for this study (212 GTR cases divided by 462 total surgical cases).

N/a, Not applicable.

Management of adult-onset craniopharyngioma also can differ between the pathological subtypes, as the discoveries of their mutational underpinnings have paved the way for targeted molecular therapies such as BRAF inhibitors (e.g., dabrafenib, vemurafenib) and MEK inhibitors (e.g., trametinib, cobimetinib) (62–64). In PCPs with confirmed BRAF V600E mutation, BRAF-targeted therapy can be used as neoadjuvant therapy to shrink tumor size (63, 65). The incorporation of these targeted agents into the therapeutic paradigm holds promise for improving outcomes, particularly in refractory or recurrent cases. There have been limited multi-participant studies evaluating response to BRAF/MEK inhibitor therapy in patients with recurrent BRAF V600E-positive PCP, although multiple case studies in adults showed response to a second, and in one study even a third, course of the same targeted therapy (66–68). This is an area that deserves particular attention for future investigation. Data on other medication options for recurrent or refractory craniopharyngiomas is limited overall, and even more so in adults. A recent case report demonstrated the use of bevacizumab (an anti-VEGF antibody) to help curb tumor growth in the setting of rapid vision changes after a failed surgical intervention for an ACP patient (69). Several case reports have been published regarding the use of tocilizumab for pediatric ACP patients (70, 71). Also in a phase 2 trial of 19 children and young adults with unresectable or recurrent craniopharyngiomas, when treated with weekly subcutaneous pegylated interferon alpha-2b for up to 18 courses median progression-free survival was 19.5 months, although none of the 11 stratum 2 patients had an objective radiographic response (72). Similar studies in an adult population would be an important avenue to explore.

Adjuvant radiation therapy (RT) is frequently employed following STR, in cases where gross surgical resection is contraindicated, or in cases of recurrent disease. Our ACP patient was offered adjuvant radiation in addition to her STR; however she opted not to pursue this out of concerns for side effects. Stereotactic RT (SRT), stereotactic radiosurgery (SRS), intensity-modulated RT and proton beam RT are predominant modalities (73–76). RT is an effective modality for providing disease control in adult patients with craniopharyngioma, both at initial diagnosis and in those with recurrent disease (77, 78); in one study of 49 adult patients with craniopharyngioma treated with either photon and proton therapy, 27 of whom were treated at initial presentation and 22 for recurrent disease, the 5- and 10-year local control rates were 100% and 94%, respectively (79). Recent studies indicate that proton beam therapy may offer reduced toxicity compared to conventional photon-based radiotherapy (80, 81). When discussing radiation treatment with our STR patient, she was most interested in pursuing proton therapy in the hope of sparing toxicity to healthy brain tissue. Ultimately though she was concerned about the limited insight into long term sequelae and additional risk posed by treatment, prompting her to hold off on proceeding. In a retrospective study of 91 adult craniopharyngioma patients, Beddok et al. found that proton therapy frequently results in late endocrinopathy, but hearing toxicity, memory impairment and visual changes were relatively rare (82). In another retrospective study of 14 adult participants who received proton therapy, Rutenberg et al. found that the 3-year local control and overall survival rates were both 100% and that there was no radiotherapy-related vision loss or optic neuropathy (83). Ultimately, further research is warranted to establish proton therapy’s definitive role in management of adult craniopharyngiomas due to the absence of comparative controlled trials in adults. Control of resistant cystic tumors can pose a particular challenge for radiation therapy and in some cases contribute to cyst expansion (84). In patients with recurrent cyst development after surgery, additional interventions including percutaneous aspiration, intracystic irradiation using beta-emitting radioisotopes such as phosphorus or yttrium, and intracystic chemotherapy (i.e., bleomycin and interferon alpha) have been successfully used in children; data is limited in the adult population (85–88).

### Related sequelae of adult-onset craniopharyngioma

**Figure 1:**
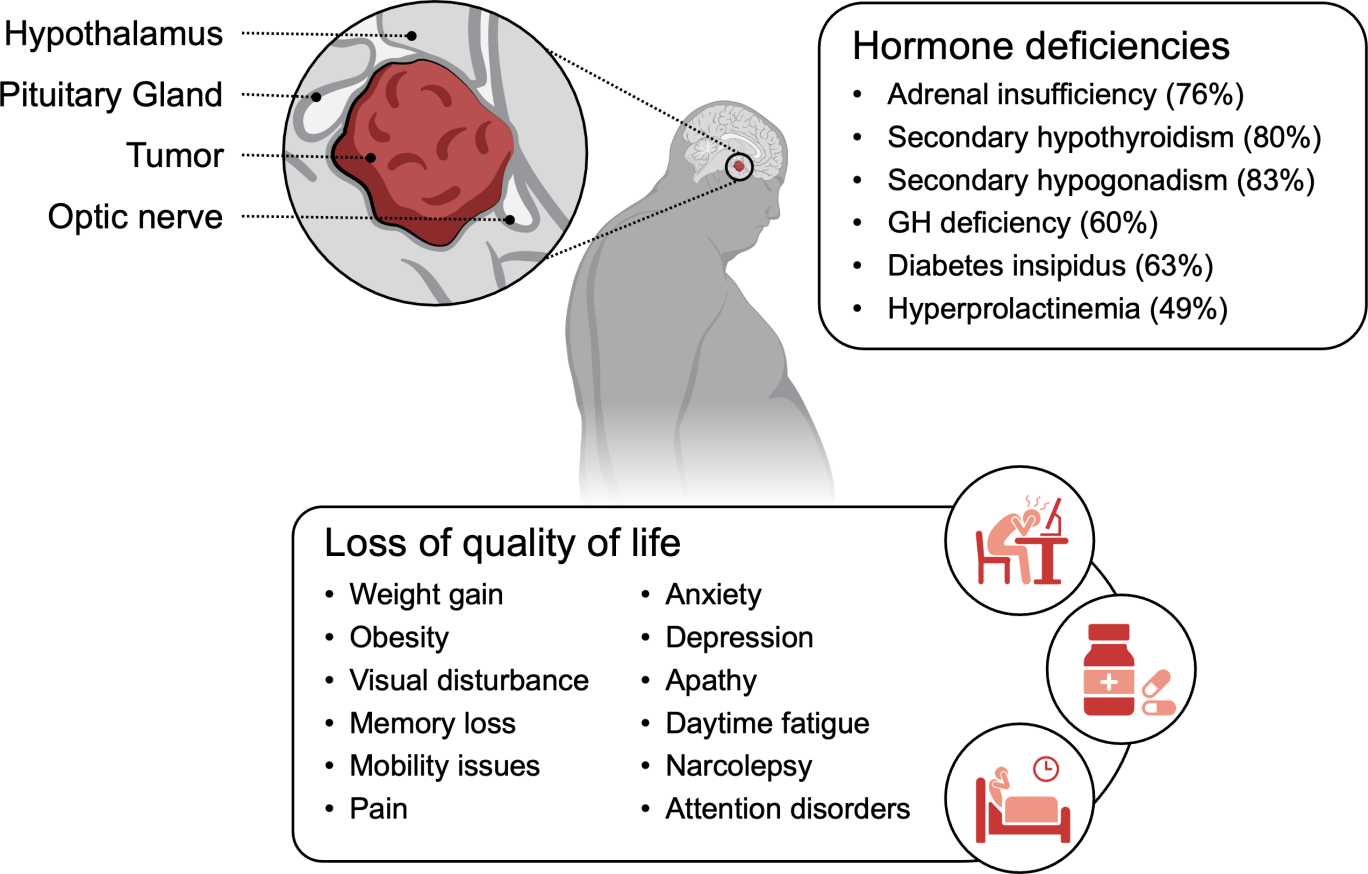
Craniopharyngioma morbidity. Common morbidities and adverse sequelae experienced by adult craniopharyngioma patients both pre- and post-treatment.

Systematic research regarding mortality and morbidity in adults is limited, with most studies looking at outcomes in pediatric craniopharyngioma patients or a mixed pediatric-adult population (89). Survival rates in these studies are reported as ranging from 89% to 94% at 5 years, 85% to 90% at 10 years and 62% to 76% at 20 years (89). One of the few adult-only retrospective cohort studies found mortality of 88% in 91 patients at 8.3 years (90). The cause of death was frequently multifactorial but rarely due to craniopharyngioma itself, with a large predominance of cardiovascular and respiratory causes of death (91). Craniopharyngioma patients have a 3- to 19-fold higher rate of cardiovascular-related mortality compared to the general population (89). At approximately 100 months after neurosurgical intervention, there was a trend toward the development of hypertension (28% at baseline to 40% at final follow up, *p* = 0.09) or need for statin therapy (30% at baseline to 43% at final follow up, *p* = 0.06) though not to a statically significant degree (90). Although craniopharyngiomas are classified as Grade I tumors, their localization in the sellar region neighboring vital structures such as the pituitary, hypothalamus and optic chiasm results in a large amount of comorbidity for patients as highlighted in **Figure 1**. Beyond symptoms noted at the time of presentation, tumor treatment itself has a high rate of complicating factors and sequelae. There currently is a dearth of adult-specific analyses, but studies thus far suggest that many of the challenges that pediatric craniopharyngioma patients face are paralleled in the adult population (4).

As previously discussed, visual disturbances are the most common presenting symptoms for adults. In turn visual preservation is an important factor to consider when treating craniopharyngioma patients. In a meta-analysis with 1307 patients, 38-42% of patients experienced some improvement in their visual disturbances following surgical intervention (92), while others demonstrated either no improvement or progression of vision loss. Fortunately for both cases above, following surgical intervention, the patients reported improvement of their visual symptoms. Natural recovery following surgery is overall difficult due to damage of the optic pathway and the limited self-renewal capacity of adult cells in this pathway. Research is ongoing regarding nerve regeneration in the hopes of improving visual function in patients who experienced permanent optic nerve damage (46).

Hormone deficiencies are another important complication to consider for both the disease itself and disease treatment, with reported rates of 54-100% in the pediatric population (46). In a retrospective study of 91 patients with adult-onset craniopharyngioma, at an average of 100 months out from neurosurgery, 76% of patients had secondary adrenal insufficiency, 80% had secondary hypothyroidism, 83% had secondary hypogonadism, 60% had GH deficiency, 63% had diabetes insipidus and 49% had hyperprolactinemia (90). Of this patient cohort, 72% had deficiencies in three or more hormones. Replacement therapy is the mainstay of treatment for this complication but can have its own difficulties with regard to ensuring appropriate titration and preventing additional complications from imprecise supplementation (90, 93). The ACP patient in case 1 required steroids, levothyroxine, and desmopression while the PCP patient in case 2 required steroids, levothyroxine, desmopressin, growth hormone, and gonadotropins.

One of the most significant implications faced by patients is weight gain or hypothalamic obesity. This is well exemplified by the ACP and PCP patients in the above cases with a BMI of 30 and 38 respectively. Following intervention, patients can experience disturbances in satiety regulation, sympathetic tone and overall decreased energy expenditure leading to morbid obesity that is largely unresponsive to conventional interventions (94, 95). As in the pediatric population, the degree of hypothalamic involvement of the mass preoperatively has been correlated with increased weight gain in adult-onset craniopharyngioma (96, 97). In a retrospective study exploring hypothalamic obesity risk factors in adults, 40.8% of patients had a weight gain of at least 5% in a median follow-up time of 12 months after initial craniopharyngioma surgery. The average weight gain was 17.59% and overall hypothalamic obesity increased from 19.2% to 29.2% following surgery. The ACP subtype was a significant risk factor for a clinically significant weight gain. Those with a higher preoperative BMI had an increased risk of hypothalamic obesity (98). A meta-analysis exploring GTR vs STR as a treatment approach in adults found that pathologic weight gain occurred in 39% and 30% of GTR- and STR-treated patients, respectively, without significant differences between the two groups (92). Interventions to overcome hypothalamic obesity are principally explored in the pediatric population (94, 99). In a case series by Zoicas et al., however, 6 adult patients with craniopharyngioma were treated with the GLP-1 agonist exenatide with notable weight loss and improved cardiovascular profiles (100). The CRANIOEXE clinical trial investigated the efficacy of exenatide in combination with intensive lifestyle interventions for weight loss in patients with craniopharyngioma related obesity; the combination was not shown to be superior when compared to placebo, although investigators saw an unexpected decrease in weight in control patients, which they felt might confound the utility of exenatide (101). Newer GLP-1s including semaglutide and tirzepatide which have found a footing in the general population for weight loss have not yet been studied in a cohort of craniopharyngioma specific patients. That being said, both our ACP and PCP patients are currently receiving semaglutide in an effort to address their obesity. In the case of our ACP patient, she has lost 30 pounds since Ozempic initiation 15 months ago while our PCP patient’s weight remains largely stable with only a 3 pound decrease since Wegovy initiation 5 months ago. Bariatric surgery has also been investigated: Bretault et al. performed a meta-analysis, noting a tendency toward weight loss, though not to a significant level. This study was limited by a small sample size (21 cases including 6 adjustable gastric banding, 8 sleeve gastrectomy, 6 roux-en-y and 1 biliopancreatic diversion cases) with a heterogeneous population that included children and adults (6 adults) (102). A study by Faucher et al. indicated less total weight reduction in craniopharyngioma related obesity patients when compared to matched controls with common obesity following bariatric surgery; total weight loss after 1 year and 5 years was lower in the craniopharyngioma group than in the control group (23.1% vs 31.4% at 1 year and 17.8% vs 26.2% at 5 years) (103). Other methods investigated in children but not yet well studied in adults to combat craniopharyngioma-associated hypothalamic obesity include sympathomimetics, octreotide, diazoxide, metformin, naltrexone, supraphysiologic T3 supplementation, GH substitution and oxytocin (94, 95, 104).

While studies suggest that adult-onset craniopharyngioma patients generally fare better than pediatric cases in terms of overall quality of life, neuropsychiatric disturbances, and memory deficits (46), significant comorbidities persist that substantially impact long-term health and well-being. One of the primary concerns is the pronounced psychosocial burden these patients face. Many years out from her original diagnosis, our ACP patient in Case 1 continues to express feelings of sadness regarding her disease, overall health, and ongoing medication burden. Despite maintaining relatively stable physical health, patients often rate their psychosocial status as markedly worse, underscoring the profound mental and emotional toll of the disease and its treatment (94). Psychological comorbidities such as anxiety, depression, and apathy are disproportionately higher in this population compared to the general public, leading to a diminished sense of well-being (94, 105). Additionally, somatic complaints are frequent, with increased reports of chronic pain and musculoskeletal issues that hinder physical mobility and contribute to overall distress (105, 106).

Moreover, neurocognitive impairments remain a prevalent challenge. Patients frequently experience daytime fatigue, narcolepsy, and attention disorders (46, 89, 94, 107). One study found that 71.5% of craniopharyngioma patients report daytime somnolence compared to 17% of controls; the majority of these cases were attributed to obstructive sleep apnea (108). These findings highlight the necessity of comprehensive, multidisciplinary care that addresses not only the physical sequelae of adult-onset craniopharyngioma but also the often-overlooked psychosocial and cognitive dimensions of the disease.

## Conclusion

In review, despite sharing many characteristics, craniopharyngioma in children and adults differ in several key aspects, including tumor type, size and associated outcomes. In children, craniopharyngiomas are almost exclusively ACP in etiology, while adults develop both ACP and PCP, with PCP being slightly more common. Pediatric craniopharyngiomas more commonly present with tumors that are larger in size, and patients more frequently endorse symptoms of increased intracranial pressure such as headaches, nausea and vomiting (6). Conversely, the most common complaint of adults is visual impairment, with only around half of patients endorsing headaches and around a sixth of patients endorsing nausea or vomiting at the time of presentation (6). Furthermore, compared with childhood ACP, adult ACP tends to be more radiologically heterogeneous with lower rates of calcification, suggesting nuanced differences in pathogenesis. While both pediatric and adult craniopharyngioma cases experience similar sequelae including visual deficits, hypothalamic obesity and hormone deficiencies, the literature varies regarding the prevalence and severity of these in the two populations (4, 9, 45, 89, 90, 96, 97). Such nuances highlight the need for age-specific approaches in diagnosis, treatment and long-term management.

When examining our two cases, both exhibit hallmark features of adult-onset craniopharyngioma. In each instance, symptoms—most notably subacute visual deterioration—prompted the patients to seek medical care. Both underwent appropriate imaging, followed by maximal safe surgical resection via transsphenoidal endoscopic endonasal surgery, and both continue to manage ongoing sequelae. Despite these similarities, each case presents distinct features and challenges. In our ACP patient, MRI findings were highly suggestive of ACP due to the mass’s pronounced calcification, whereas the PCP patient’s diagnosis based on imaging alone was not completely clear cut and was further confounded by his prior erroneous diagnosis of Rathke’s cleft cyst based on histology. Additionally, the ACP tumor’s proximity to the hypothalamus and mammillary bodies, coupled with the inherently adherent nature of ACP to proximal tissue, made gross total resection (GTR) technically difficult, necessitating a subtotal resection (STR) instead. In contrast, our PCP patient underwent an uncomplicated GTR. Following STR, our ACP patient was offered adjuvant radiation therapy, including proton therapy, which allows for more targeted radiation delivery. However, due to concerns about potential risks, she declined further treatment. Fortunately, neither patient has experienced tumor progression or recurrence to date. Nevertheless, both continue to grapple with significant daily challenges, particularly in managing sequelae such as hypothalamic obesity and hormone replacement for panhypopituitarism. The two patients remain on hormone supplementation, requiring frequent symptom monitoring and lab checks. Regarding obesity, while our ACP patient has achieved notable success in losing weight with semaglutide, the same approach has not been as effective for our PCP patient. From a psychosocial standpoint however, our ACP patient appears to be struggling more, expressing greater dissatisfaction and sadness regarding her condition.

The past decade has brought considerable advances in the realm of adulthood craniopharyngioma, as well as craniopharyngioma management as a whole. It was only 2014 when investigators first noted that the PCP subtype carried the BRAF V600E mutation, which has paved the way for further diagnostic testing as well as targeted immunotherapy treatments that are undergoing current assessment. Rapid improvements in imaging protocols, radiomics, artificial intelligence and molecular testing hold the potential for expedited and improved craniopharyngioma diagnosis and monitoring. Regarding treatment, the validation and adoption of endoscopic endonasal surgery techniques in the adult cohort, ongoing assessment of radiation techniques such as photon therapy or intracystic radiation and aforementioned immunotherapies hope to improve tumor control as well as complicating sequelae. While many studies investigating how to manage the sequelae themselves, such as hypothalamic obesity, are limited to the pediatric population, GLP-1 agonists have shown promise in adults for this obstinate comorbidity (100). Ultimately, in the coming years, we will learn the utility of these novel techniques and see how innovative thinking may further improve the standard of care for craniopharyngioma patients.
